# Examination of Transition Readiness, Medication Adherence, and Resilience in Pediatric Chronic Illness Populations: A Pilot Study

**DOI:** 10.3390/ijerph17061905

**Published:** 2020-03-15

**Authors:** Tanvi Verma, Jennifer Rohan

**Affiliations:** 1 Children’s Hospital of Richmond, Virginia Commonwealth University, Richmond, VA 23219, USA; 2 Cancer Prevention and Control Program, Massey Cancer Center, Virginia Commonwealth University, Richmond, VA 23219, USA

**Keywords:** resilience, transition, adherence, pediatrics, chronic illness

## Abstract

The present study assessed the relationship between resilience, adherence, and transition readiness in adolescents/young adults with chronic illness. Participants included 50 patients (Mean age, *Mage* = 17.3 ± 2.1 years) diagnosed with an oncology disorder (*n* = 7; 12.1%), hematology disorder (*n* = 5; 8.6%), nephrology disorder (*n* = 31; 53.4%), or rheumatology disorder (*n =* 7; 12.1%). Patients were administered questionnaires assessing resilience (Conner–Davidson Resilience Scale 25-item questionnaire, CD-RISC-25), transition readiness (Self-Management and Transition to Adulthood with Rx=Treatment, STARx), and self-reported medication adherence (Medication Adherence Module, MAM). Medical chart reviews were conducted to determine objective medication adherence rates based on pharmacy refill history (medication adherence ratios). A multivariate correlation analysis was used to examine the relationship between resilience, transition readiness, and adherence. There was a moderate relationship (*r* = 0.34, *p ≤* 0.05) between resilience (*M* = 74.67 ± 13.95) and transition readiness (*M* = 67.55 ± 8.20), such that more resilient patients reported increased readiness to transition to adult care. There also was a strong relationship (*r* = 0.80, *p ≤* 0.01) between self-reported medication adherence (*M* = 86.27% ± 15.98) and pharmacy refill history (Mean Medication Adherence Ratio, *M_MAR_* = 0.75 ± 0.27), which indicated that self-reported adherence was consistent with prescription refill history across pediatric illness cohorts. Our findings underscore the importance of assessing resilience, transition readiness, and adherence years before transitioning pediatric patients to adult providers to ensure an easier transition to adult care.

## 1. Introduction

Children, adolescents, and young adults with a chronic disease face multiple challenges across the duration of their illness, including the multifaceted daily challenges they face when managing a chronic illness. These include taking daily medications as prescribed, adhering to complex treatment regimens, and engaging in healthy lifestyle changes (e.g., dietary changes, increased physical activity, managing functional limitations, etc.) [1,2,3]. It is the ability to overcome these challenges, including following complex medical recommendations and successfully managing their chronic illness, which leads to successful disease management and ultimately successful transition from pediatric to adult care [1,2,4,5,6,7,8,9,10,11,12,13,14]. Improving self-management and health behaviors while preparing adolescents for a successful transition from pediatric to adult care remains a critical area of need to minimize the short-term and long-term consequences of poor self-management behaviors on health outcomes. 

Resilience or the ability to bounce back when faced with adversities has been shown to correlate with transition readiness and disease outcomes [9,15,16,17]. The concept of resilience emerged in the mid-1970s when psychologists recognized that certain individuals were better able to overcome stressors such as life conflicts, health problems, and trauma compared to other peers [17,18,19,20,21,22,23]. Resilience is characterized by five key components: perseverance, having a balanced perspective, the belief that life is meaningful, self-reliance, and the belief in individuality [9,15,16,17,24,25]. The relationship between resilience, treatment adherence, and transition readiness has not been fully explored in an adolescent and young adult chronic illness population framework, and hence remains a critical area of investigation in both research and clinical care domains. The present study will explore the relationship between resilience, adherence, and transition readiness across multiple pediatric chronic illness cohorts. 

Children with chronic illness often face adversity due to the complex nature of managing a chronic illness. Fenton et al. found that adolescents have low transition readiness when they experience significant negative health outcomes [11]. Examples of factors that result in negative health outcomes include participation in invasive procedures, medical interventions, and functional limitations as a result of their disease (e.g., limited physical activity, inability to participate in sports, increased school absences), as well as increased psychological distress such as feelings of inferiority, insecurity, and injustice [1,2,3,17,18,20,23]. Therefore, there is a need to examine the extent to which factors producing negative health outcomes affect transition readiness. During adolescence, patients often take increasing responsibility for their disease management and must adapt to the unique challenges associated with adolescence while also managing a complex treatment regimen such as taking multiple medications and engaging in healthy lifestyle changes [9,11,26,27,28]. During this critical period of development, adolescents with chronic illness are also faced with the need to transition from pediatric to adult care, which can be exciting but also frightening at the same time as they transition to a whole set of new providers while leaving their pediatric providers who they have known for many years [7,11,12,14]. 

Resilience is a measure of adaptability to stress and ability to overcome challenges [9,15,16,17,24,25]. Therefore, it follows that patients with higher resilience will likely be better equipped to overcome the challenges presented by their disease, including being able to successfully take charge of their own care, which will ultimately lead to successful transition from pediatric to adult care [1,2,9,29]. Therefore, it is likely that higher resilience will be associated with better adherence rates and readiness to transition to adult care. In contrast, low healthcare transition readiness will likely be associated with lower medication adherence [1,2,29]. The present study is a cross-sectional pilot study which assessed resilience, medication adherence, and transition readiness in a diverse sample of adolescents and young adults diagnosed with a chronic illness. It is hypothesized that there will be a moderate to strong relationship between resilience, medication adherence, and transition readiness, such that patients with higher resilience will also have better medication adherence and higher transition readiness scores. 

## 2. Methods

### 2.1. Study Design and Participants

To our knowledge, this is the first study that investigated the relationship between resilience, medication adherence, and transition in a diverse sample of adolescent and young adult patients with a chronic illness. Patients between the ages of 13 and 24 years who were diagnosed with a chronic illness requiring daily medications were enrolled in a single site cross-sectional pilot study. All patients were followed by a subspecialty clinic (hematology–oncology, nephrology, or rheumatology) in an academic medical center. 

Patients were eligible for study participation if they were currently living with a chronic disease, having been diagnosed for at least 3 months and requiring regular medical visits to a subspecialty clinic. Patients in this study took at least one daily medication for their chronic disease as recorded in their self-reported medication list and pharmacy refill records, and were able to read and understand English at a 5^th^ grade level. Patients who were acutely ill at the time of their clinic visit were excluded from study participation to reduce potential burden of research participation. The study protocol was reviewed and approved by the Institutional Review Board (IRB). All youth provided written or verbal assent to participate and their caregivers provided written informed consent/parental permission. In the present study, researchers identified 65 eligible participants who were approached by research staff. Of the 65 eligible participants, 50 (76.92%) agreed to participate in the study. Patients received incentives for their participation (e.g., $20 cash). 

### 2.2. Measures

Demographic and Clinical Data: Demographic and clinical data were collected via self-report. Medical data were collected via medical chart review. 

Conner–Davidson Resilience Scale (CD-RISC): The CD-RISC is a self-report, 25-item questionnaire that measures unique aspects of resiliency using a 5-point Likert scale ranging from 0 = *“not at all”* to 4 = *“true nearly all the time”* [30]. The full scale takes about 10 minutes to complete. Total scores range from 0 to 100 with higher scores reflecting greater resiliency. In the general population, the mean of the CD-RISC is 80 with an interquartile range of 73–90 [30,31,32,33]. This measure has good reliability and validity and has been validated in adolescents and young adults in a number of settings [30,31,32,33]. Internal consistency was assessed using Cronbach’s α and was 0.91.

Transition Readiness Scale: The Self-Management and Transition to Adulthood with Rx=Treatment (STARx) questionnaire is an 18-item self-report questionnaire that measures overall transition readiness in adolescent and young adult participants [10]. The questionnaire takes approximately three minutes to complete. Participants rate each item on a 5-point Likert scale (0 = *“never”* to 4 = *“always”*) with total scores ranging from 0 to 90. Higher scores correspond to higher transition readiness [10]. This measure is well-validated [10]. Internal consistency was assessed using Cronbach’s α and was 0.74. 

Medication Adherence Module (MAM): The MAM is a semi-structured tool with four modules that assess adherence across multiple domains (i.e., medication, diet, exercise, and clinic attendance) [34,35,36]. For this study, only the medication module was used given the specific interest in medication adherence. Participants’ knowledge of medication regimens was assessed using an interview format (e.g., interviewers asked patients to provide the name, purpose, and dosing for each prescribed medication), and self-reported adherence was obtained (i.e., self-report of the number of medications taken as prescribed vs. number of medication doses that were missed). A missed adherence score (MAS) or nonadherence was generated by dividing the number of missed doses by the total number of doses prescribed and multiplying this number by 100%. The present study defined poor adherence as a MAS score of 80% or lower, which was based on previous research [34,35,36]. Internal consistency was assessed using Cronbach’s α and was 0.83. 

Pharmacy Refill Records: Pharmacy refill records were used to assess objective medication adherence rates. Medication refill records were obtained by examining the pharmacy refill records contained within the electronic medical record. Refill records were obtained for the three medication fills prior to study participation. A medication adherence ratio (MAR) was calculated as follows: MAR = *number of days supplied/number of days observed during the time interval*. Lower MAR indicated a higher number of days in which participants did not take their medication as prescribed, resulting in worse medication adherence. Poor adherence was defined as a MAR of 0.8 or lower, which was based on previous research [37]. 

### 2.3. Data Analytic Plan

Descriptive statistics were generated for demographic and clinical data, transition readiness, medication adherence metrics, and resilience. A multivariate correlation analysis was used to compare the relationship between transition readiness, resilience, and medication adherence.

## 3. Results

### 3.1. Description of Sample

The demographic and medical characteristics of the sample (*n* = 50) are presented in Table 1. At baseline, the mean age of the sample was 17.3 years (range: 13–22 years). The majority of the sample was diagnosed with a nephrology disorder (62%); however, 24% were diagnosed with a hematology–oncology disorder. The majority of the sample was of non-Hispanic, African American ethnicity/race (60%) with the majority being female (58%). 

### 3.2. Multivariate Correlation Analysis

**Figure 1:**
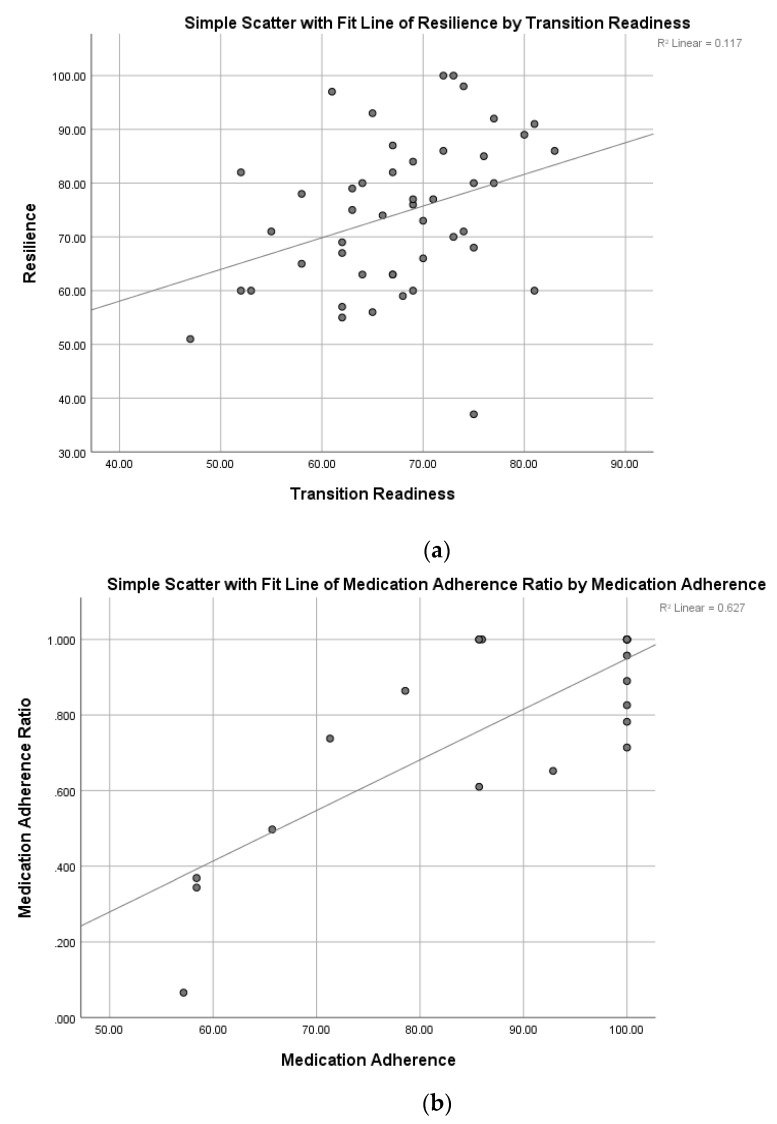
(**a**) Relationship between resilience and transition readiness. (**b**). Relationship between multiple measures of medication adherence.

Correlations are shown in Table 2. There was a moderate relationship (*r* = 0.34) between resilience and transition readiness scores, such that more resilient patients reported an increased readiness to transition to adult care (see Figure 1a). There also was a strong relationship (*r* = 0.79, *p ≤* 0.01) between self-reported medication adherence and medication adherence ratio scores based on pharmacy refill history. Prescription refill history is an objective measure of medication adherence. Higher adherence rates as demonstrated by prescription refill history were consistent with higher self-reported adherence rates (see Figure 1b).

**Figure 2:**
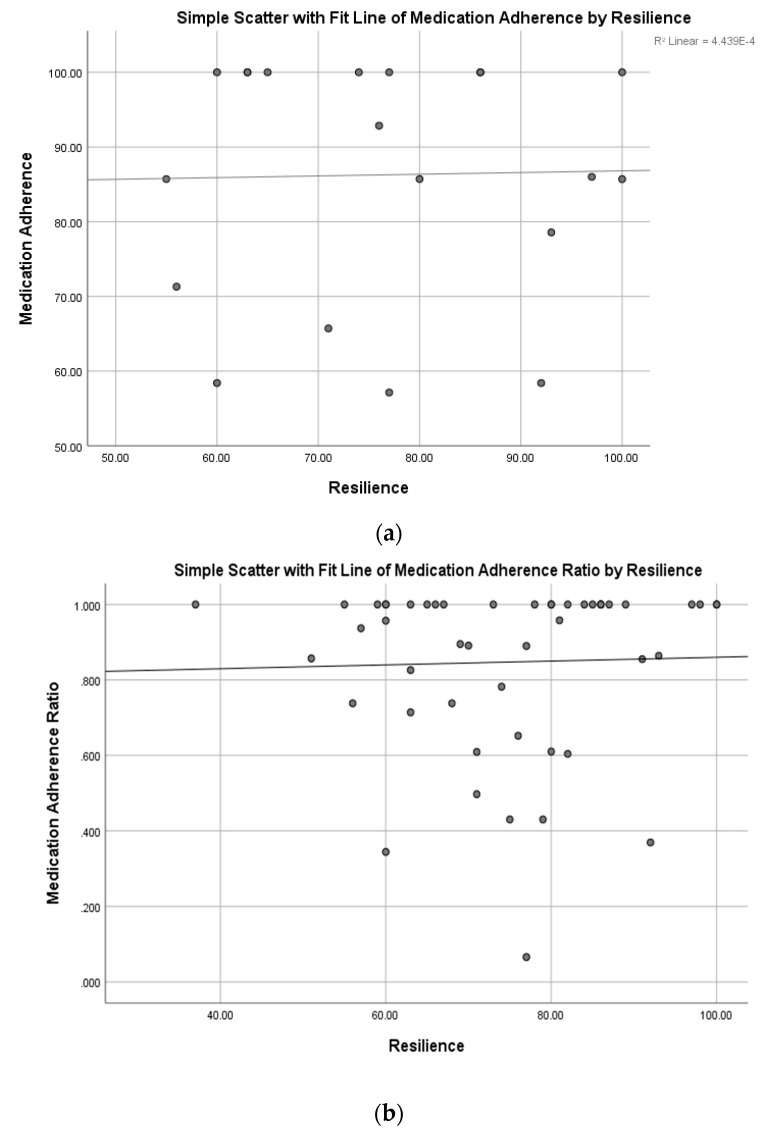
(**a**) Relationship between resilience and self-reported medication adherence. (**b**) Relationship between resilience and medication adherence ratios.

**Figure 3:**
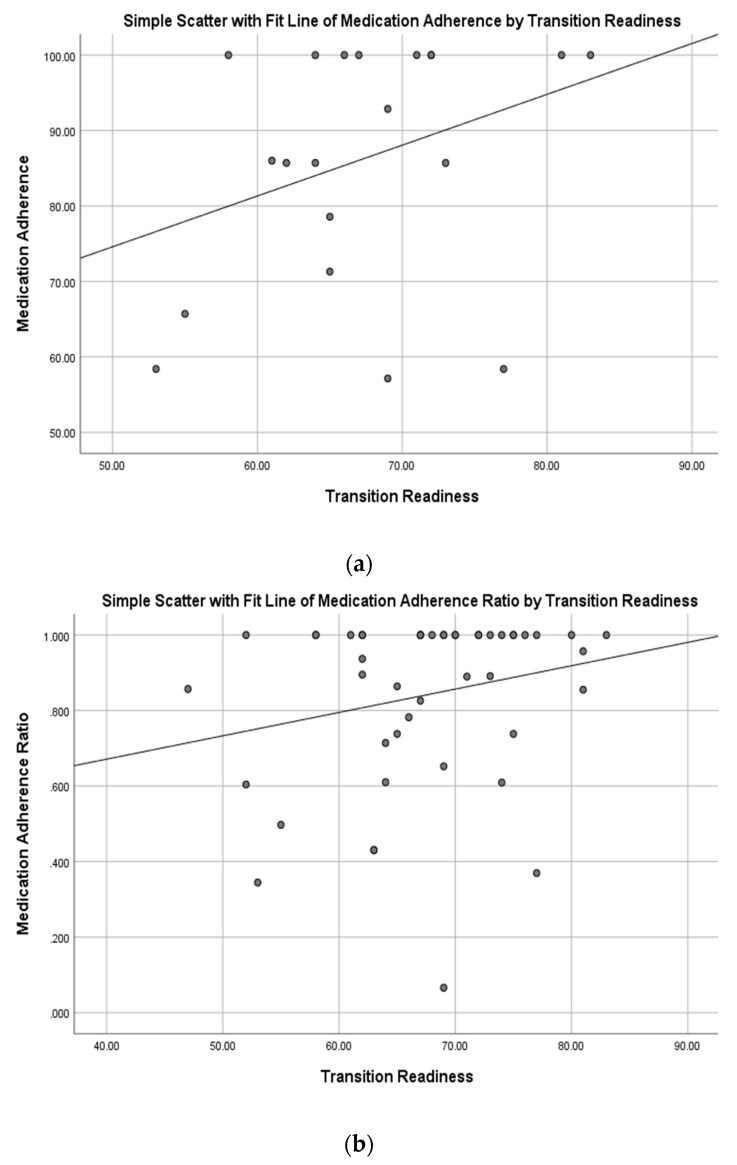
(**a**) Relationship between transition readiness and self-reported medication adherence. (**b**) Relationship between transition readiness and medication adherence ratios.

There was not a significant relationship between self-reported medication adherence rates and resilience (*r* = 0.02; Figure 2a), resilience and medication adherence ratios (*r* = 0.03; Figure 2b), transition readiness and self-reported adherence (*r* = 0.33; Figure 3a), or transition readiness and medication adherence ratios (*r* = 0.22; Figure 3b). 

### 3.3. Regression Analysis

Linear regression analyses were conducted to evaluate the relationship between resilience and transition readiness. Resilience significantly predicted transition readiness (β = 0.342, R2 = 0.098, *p* = 0.018), which suggested that more resilient individuals reported an increased readiness to transition from pediatric to adult care.

### 3.4. Mediation Analysis

Regression analysis was used to investigate a mediation effect of medication adherence (prescription refill history) on resilience and transition readiness. The outcome variable for this analysis was transition readiness (CD-RISC-25). The predictor variable was resilience (STARx). The mediator variable was medication adherence (MAR). The indirect effect of resilience on transition readiness was not found to be statistically significant (effect = 0.0019, 95% C.I. (–0.0281, 0.0519)). The indirect effect was tested using a percentile bootstrap estimation approach with 10,000 samples, implemented with the PROCESS macro Version 3 [38]. Thus, medication adherence as measured by pharmacy refill data did not mediate the relationship between resilience and transition readiness.

## 4. Discussion

To our knowledge, this is the first study that described the relationship between transition readiness, resilience, and medication adherence in a diverse cohort of adolescent and young adult patients with chronic illness. Consistent with hypotheses, the present study demonstrated a moderate relationship between transition readiness and resilience, such that those who were more resilient were more likely to report increased readiness for transitioning to adult care. Despite finding a significant relationship between self-reported adherence and objective medication adherence rates (based on pharmacy refill data), the present study did not find a significant relationship between medication adherence and resilience, or between medication adherence and transition readiness, which suggests that resilience and transition readiness factors do not negatively or positively influence self-reported or objective measures of medication adherence. In fact, as shown in Figure 2a, there is a relatively stable relationship between resilience and self-reported medication adherence. In contrast, the medication adherence ratio is higher for patients with higher self-reported resilience scores (Figure 2b), although this finding was not significant. Similarly, the relationship between self-reported medication adherence and transition readiness (Figure 3a) and the relationship between objective measures of medication adherence (pharmacy refill data, Figure 3b) were both relatively stable with nonsignificant relationships observed. 

This study serves as a basis for future research investigating modifiable factors known to affect resilience, medication adherence, and transition readiness in children and adolescents with chronic illness. Results of this pilot study suggest that there was a strong relationship between resilience and transition readiness, and between multiple measures of medication adherence across pediatric chronic illness cohorts. Future research should incorporate a longitudinal study to examine patterns of medication adherence, resilience, and transition readiness over time, including during the critical period of transition from pediatric to adult care. 

Although the present study did not investigate the relationship between psychological distress, resilience, transition readiness, and adherence, future research should investigate the inter-relationship between these important and often inter-related variables. It is well-documented that resilient individuals have lower rates of depression, anxiety, and mood symptoms, and higher quality of life. That said, children, adolescents, and young adults with chronic disease often report lower health-related quality of life across multiple domains, including physical, emotional, social, and school domains [1,2,39,40]. Additionally, there are higher rates of depression observed in pediatric, adolescent, and young adult patients with chronic illness, which is often associated with higher rates of medication nonadherence and worse resilience over time [29,41,42,43,44,45,46,47]. Furthermore, previous research has shown that depressive symptoms often correlate with lower quality of life and increased psychological distress [29,41,42,45,46,47,48]. Knowing the relationship between psychological distress, quality of life, resilience, transition readiness, and medication adherence will allow clinicians and researchers to develop more clinically effective interventions targeted at improving resilience and psychological functioning. Our findings underscore the importance of investigating novel interventions focused on building resilience within pediatric patients diagnosed with chronic conditions, which will enhance their readiness to transition to adult care.

There are several limitations that should be considered when interpreting the present findings. The current study is a cross-sectional, pilot study with a small sample size of 50 patients diagnosed with a chronic illness. Future research should examine a more diverse sample of diagnoses with a larger sample size between illness cohorts, which are studied over a longitudinal time period. Another limitation is the relatively homogenous sample of patients. The majority of patients identified as non-Hispanic, African American ethnicity/race (60%) and were diagnosed with nephrology disorders (62%). Generalizability of the current findings should be established with a more diverse patient sample studied over a longer time frame. 

The findings of the current study have important implications for future research and for clinical care. Our findings underscore the importance of developing tailored and personalized interventions targeted at increasing resilience in children and adolescents, which will ultimately enhance their transition from pediatric to adult care. Furthermore, our findings can provide an important foundation for future research designed to investigate the relationship between psychological distress, resilience, health behaviors, and transition readiness. Previous research has demonstrated that resilience is negatively associated with neuroticism, i.e., patients with low resilience will be prone to experiencing negative emotions, poor coping styles, and difficulty controlling impulses [49]. While resilience is dependent on temperament and personality, resilience also is dependent on skills such as problem solving, and often is associated with the tendency to use task-oriented coping strategies [24,49]. The findings from this pilot study and future research can collectively be used to develop patient-centered and cost-effective interventions that can be easily incorporated into real-world clinical settings, including utilization of m-Health, telehealth, and/or in-person interventions. 

What makes resilience an attractive area for study is that it can be modified and improved with a targeted intervention, such as acceptance and commitment therapy and/or cognitive behavioral therapy [18,19,20,22,23,24,50,51]. Interventions designed to enhance resilience have been shown to reduce and prevent depression, reduce hopelessness, and reduce and prevent anxiety [23,24,50]. Future research should examine the impact of potentially modifiable individual, family, and cultural factors associated with medication adherence, resilience, and transition readiness in chronic illness, which will further enhance development of novel interventions. 

## 5. Conclusions

The present study, a cross-sectional pilot study, assessed the relationship between resilience, adherence, and transition readiness in adolescents/young adults with chronic illness. Consistent with hypotheses, there was a significant relationship between self-reported adherence and objective medication adherence rates. However, the present study did not find a significant relationship between medication adherence and resilience, or between medication adherence and transition readiness, which suggests that resilience and transition readiness factors do not negatively or positively influence self-reported or objective measures of medication adherence. Ultimately, our findings, in combination with future research, could provide healthcare providers with the necessary tools to promote healthy behaviors and resilience, which might ultimately enhance the transition from pediatric to adult care, and reduce the short-term and long-term adverse outcomes associated with psychological distress, low transition readiness, and poor resilience. 

## Figures and Tables

**Table 1:** Demographic and medical characteristics of baseline sample (*n* = 50).

Child’s/adolescent’s age at baseline (years), M ± SD (range)	17.3 ± 2.1 years (13.1–22.6 years)
Type of diagnosis, *n* (%)	
Nephrology	31 (62%)
Rheumatology	7 (14%)
Hematology	5 (10%)
Oncology	7 (14%)
Duration of diagnosis (years), M ± SD (range)	6.15 ± 5.0
Child’s gender, *n* (%)	
Male	21 (42%)
Female	29 (58%)
Child’s ethnicity/race, *n* (%)	
Non-Hispanic, Caucasian	17 (34%)
Non-Hispanic, minority	30 (60%)
Hispanic	3 (6%)
Scored measures (M ± SD)	
Resilience	74.67 ± 13.95
Transition readiness	67.55 ± 8.20
Medication adherence	86.27% ± 15.98
Medication adherence ratio (pharmacy refill)	0.84 ± 0.23

**Table 2:** Correlation matrix for resilience, transition readiness, and medication adherence across pediatric chronic illness populations.

	Resilience	Transition Readiness	Self-Reported Medication Adherence	Medication Adherence Ratio
**Resilience**	1.00			
**Transition readiness**	0.34 *	1.00		
**Self-reported medication adherence**	0.02	0.33	1.00	
**Medication adherence ratio**	0.03	0.22	0.79 **	1.00

Note: * *p* ≤ 0.05; ** *p* ≤ 0.01.
